# Stress and growth biomarkers in adult rainbow trout (*Oncorhynchus mykiss*) reared in a novel photo-electrocatalytic recirculating aquaculture system

**DOI:** 10.3389/fvets.2026.1888391

**Published:** 2026-10-01

**Authors:** Elisa Fonsatti, Martina Bortoletti, Lisa Maccatrozzo, Laura Gregoletto, Elena Negrato, Carlo Poltronieri, Nadia Cherif, Tarek Temraz, Giorgio Mirra, Alessia Di Giancamillo, Annamaria Costa, Gian Luca Chiarello, Giuseppe Radaelli, Daniela Bertotto

**Affiliations:** 1 Department of Comparative Biomedicine and Food Science, University of Padova, Legnaro, Italy; 2 Aquaculture Laboratory, National Institute of Sea Sciences and Technologies, Salammbo, Tunisia; 3 Marine Science Department, Canal Suez University, Ismailia, Egypt; 4 Department of Biomedical Sciences for Health, University of Milano, Milano, Italy; 5 Department of Veterinary Medicine and Animal Sciences, University of Milano, Lodi, Italy; 6 Department of Chemistry, University of Milano, Milano, Italy

**Keywords:** gene expression, photoelectrocatalysis, rainbow trout, RAS, stress biomarkers

## Abstract

**Introduction:**

The present study is part of the PRIMA Fish-PhotoCAT project, which aims to evaluate the effectiveness of a smart purification system for improving water quality, fish production, and environmental sustainability in aquaculture. The system employs an innovative TiO_2_-based photo-electrocatalytic (PEC) filter that combines the photocatalytic properties of TiO_2_ with the biocidal effect of UV radiation to remove nitrogen compounds from rearing water.

**Methods:**

Adult rainbow trout were reared at low (LD, 15 kg/m^3^) and high (HD, 30 kg/m^3^) stocking densities for four and two weeks, respectively. Three tanks equipped with conventional mechanical, biological, and UV filtration served as controls (CTRL), while three tanks were fitted with the PEC treatment unit (PEC). Plasma, skin mucus, muscle, fins, and scales were collected for cortisol determination by radioimmunoassay. Plasma total proteins (TP), glucose, and lactate were measured as secondary stress indicators. Oxidative stress was assessed through Advanced Oxidation Protein Products (AOPP) and the AOPP/TP ratio. Hepatic expression of *Insulin-like Growth Factor 1* (IGF-I), *Heat Shock Protein 70* (HSP70), and *Cytochrome P450*, *family 1, subfamily A, polypeptide 1 (CYP1A1)* was quantified by real-time PCR.

**Results:**

Cortisol concentrations in all sampled matrices did not differ significantly between CTRL and PEC groups at either stocking density. Similarly, plasma glucose, lactate, and TP remained unchanged between treatments. Hepatic expression of IGF-I, HSP70, and CYP1A1 also showed no significant differences. The AOPP/TP ratio was comparable between groups, although AOPP concentrations were higher in the PEC group under HD conditions. Despite this increase, cortisol values remained within physiological ranges, indicating no evidence of marked stress.

**Discussion:**

The PEC water treatment system provided rearing conditions comparable to conventional filtration with respect to physiological, biochemical, and molecular stress responses in adult rainbow trout. The absence of treatment-related changes in cortisol, metabolic indicators, and stress-related gene expression suggests that PEC technology does not compromise fish welfare under the tested conditions. However, the increase in AOPP observed at high stocking density indicates a potential oxidative response that deserves further investigation. Overall, these findings support the use of PEC technology as a sustainable water treatment strategy for aquaculture while maintaining fish health and welfare.

## 1 Introduction

A general objective of European policies is to promote the environmental sustainability of agricultural production, and various strategies and guidelines have emphasized the need for sustainable food production to achieve climate neutrality by 2050 while reducing the environmental impact of animal farming (58). Within this framework, aquaculture has emerged as a relevant food production system, playing a central role in global food supply (Naylor et al., 2023). Aquaculture is rapidly expanding (59), and its growth requires continuous improvements to further enhance sustainability and reduce environmental impact. In this context, different farming strategies and methods are being considered to improve aquaculture sustainability, and among these, recirculating aquaculture systems (RAS) are emerging as a promising approach. In fact, RAS can reduce water consumption by up to 90–99% (3) and lower environmental impact through continuous water treatment rather than water exchange (4). A key aspect to consider when discussing RAS is water quality, as recirculating water needs to be treated to avoid the accumulation of toxic compounds; in particular, fish protein catabolism leads to ammonia production, which is highly toxic, especially in its non-ionized form (NH₃), and represents a major limiting factor in aquaculture systems (5, 6). Water quality is a critical determinant of fish welfare, since the accumulation of nitrogen-containing compounds, particularly ammonia, can impair growth, physiological functions, and survival in aquatic organisms (7, 8). To manage and control ammonia accumulation, different remediation strategies are employed in aquaculture systems, including physico-chemical methods and biological nitrification processes (9, 10). The use of photocatalysis as an advanced oxidation process for ammonia removal has been employed and has been successfully tested in aquaculture systems (11–13). More recently, photoelectrocatalytic (PEC) systems have been developed to improve water remediation performance and ammonia conversion efficiency by combining TiO_2_-based photocatalysis with an external electrical bias (14–16). These technologies are designed to improve water quality and fish conditions while lowering the environmental impact; however, assessing their impact on physiological and stress-related responses is essential to understand their potential effects on fish welfare.

The stress response in fish is typically divided into three phases: primary, secondary, and tertiary. Exposure to a stressor activates the hypothalamic–pituitary–interrenal (HPI) axis, leading to an increase in circulating cortisol levels (17). Cortisol is considered a key biomarker of the primary stress response, reflecting acute stress in plasma and mucus due to its rapid increase, and chronic stress due to its accumulation in other tissues such as fin and scales (18–20). The activation of the hypothalamic–pituitary-interrenal (HPI) neuroendocrine axis also leads to the release of growth hormone (GH) by the pituitary gland, which stimulates the production of Insulin-like Growth Factor I (IGF-I) in target organs such as the liver and muscle (21). However, prolonged cortisol elevation can negatively affect growth performance by modulating the GH/IGF axis (22) and altering the expression of growth-related genes, including IGF-I (23, 24, 60). An increase of cortisol levels in blood also lead to elevated glucose levels in plasma (25), while stress condition can induce increased muscular activity, activating anaerobic glycolysis and leading to lactate production (26), making glucose and lactate useful markers of the secondary stress response. Moreover, stressors can induce, at the cellular level, the synthesis of Heat Shock Proteins (HSPs), a conserved group of molecular chaperones that are involved in cellular homeostasis. Among these, in fish, HSP70, is upregulated in response to environmental challenges (23, 27, 28). The Cytochrome P450 (CYP) enzyme system represents a useful molecular biomarker due to its role in the response to environmental contaminants and the maintenance of physiological homeostasis; in particular, CYP1A1 is used to assess exposure and response to environmental stressors (29). Stress conditions can also enhance the production of reactive oxygen species (ROS), potentially leading to oxidative damage; among the related biomarkers, advanced oxidation protein products (AOPP) are commonly used to evaluate protein oxidation and systemic oxidative stress (24, 30, 31).

The aim of the present study is to evaluate the effects of a photoelectrocatalytic (PEC) system integrated into a RAS on stress- and welfare-related responses in adult trout reared at two different stocking densities, through a combined molecular and physiological approach.

## 2 Materials and methods

### 2.1 Experimental design, fish rearing, and sample collection

Adult rainbow trout (*Oncorhynchus mykiss*) were obtained from a commercial farm (Foglio Angelo, S.S. Bagolino, Italy) and acclimatized for 1 month prior to the experimental trial. Fish were then randomly distributed into six independent 500-L tanks at the experimental facility of the Spallanzani Institute (Cremona, Italy).

The tanks were divided into two experimental groups: three control tanks (CTRL), equipped with a conventional water treatment system including mechanical filtration, biological filtration, and UV disinfection, and three tanks fitted with a photoelectrocatalytic (PEC) water treatment unit. The PEC system integrates UV irradiation with an electrocatalytic process based on a TiO_2_-coated titanium mesh, as previously described (32, 33). Fish were maintained under a natural photoperiod and fed twice daily at 3% of body weight using a commercial diet (Nutria, Skretting Italia S.p.A., Mozzecane (VR), Italy), in accordance with EFSA/FAO recommendations (Commission Regulation 889/2008).

Fish were reared under two stocking density conditions: low density (LD, 15 kg/m^3^, IBW: 156 ± 4.5 g) for 4 weeks and high density (HD, 30 kg/m^3^, IBW: 159 ± 5.2 g) for 2 weeks. At the end of the trial, fish (LD *n* = 90, HD *n* = 60) were euthanized with an overdose of ethyl 3-aminobenzoate methanesulfonate (MS222, 300 mg/L; Sigma-Aldrich, Milan, Italy) according to standard ethical procedures. The shorter exposure period adopted for the HD trial was selected as a precautionary measure to minimize the duration of exposure to a potentially more stressful husbandry condition, as high stocking density is recognized as a relevant chronic stressor in rainbow trout (34, 35). The low- and high-density experiments were conducted as two independent trials designed to evaluate the effects of the PEC system under two different rearing conditions. Blood, skin mucus, muscle, fin, scales and liver were collected. A subset of fish was used for the different analyses. Specifically, in the low-density trial, plasma cortisol, glucose, and AOPP were determined in 30 fish (15 CTRL and 15 PEC), whereas gene expression analyses were performed on 12 fish (6 CTRL and 6 PEC). In the high-density trial, plasma cortisol, glucose, and AOPP were determined in 20 fish (10 CTRL and 10 PEC), while gene expression analyses were performed on 10 fish (6 CTRL and 4 PEC). Blood samples were collected from the caudal vein using 2.5 mL syringes and transferred into heparinized tubes. Samples were centrifuged at 4 °C (5,000×*g* for 10 min) to obtain plasma, which was subsequently stored at −20 °C until analysis. Skin mucus was collected by scraping the side of the fish with a plastic rod. Scales were obtained by gently scraping the skin surface below the first dorsal fin using a scalpel, while the upper lobe of the caudal fin was excised with scissors. In addition, liver samples were collected and preserved in RNAlater solution (Invitrogen by ThermoFisher™, Waltham, Massachusetts, USA) at 4 °C overnight, and then stored at −20 °C until further analyses. All collected tissues were maintained at −20 °C until processing.

The trial was conducted in agreement with the Italian regulations on animal experimentation and ethics (DL 26/2014) and the European regulation (Dir. 2010/6). The trial was approved by the Animal Welfare Body of the University of Milan (number 62_2023).

### 2.2 Water quality assessment

Water quality parameters were monitored throughout both experimental trials as part of the companion studies describing the low-density and high-density experiments (36). Briefly, water temperature, dissolved oxygen, and pH were monitored daily, whereas ammonia, nitrite, and nitrate concentrations were measured twice weekly. Titanium release into the water was assessed by inductively coupled plasma mass spectrometry (ICP-MS). A summary of the main physicochemical parameters is reported in the Results section to provide the environmental context for the biological responses investigated in the present study, whereas the complete methodological details and datasets have been previously published (36).

### 2.3 Radioimmunoassay (RIA)

Cortisol was extracted from plasma, fin, and scales samples as described in (20). Skin mucus samples were also used for cortisol determination. Mucus was analyzed without prior extraction and, when necessary, diluted in PBS (1:2 or 1:4). Aliquots of 10–20 μL were then used for radioimmunoassay (RIA).

Cortisol concentrations in plasma, mucus, fin and scale extracts were quantified using a microplate radioimmunoassay (RIA), following the protocol described by Bertotto et al. (18) and Meloni et al. (20). The sensitivity of the assay, defined as the hormone dose corresponding to 90% binding (B/B₀), was 1.56 pg./well. The anti-cortisol serum exhibited the following cross-reactivities: cortisol 100%, prednisolone 44.3%, 11-deoxycortisol 13.9%, cortisone 4.95%, corticosterone 3.5%, prednisone 2.7%, 17-hydroxyprogesterone 1.0%, 11-deoxycorticosterone 0.3%, dexamethasone 0.1%, progesterone <0.01%, 17-hydroxypregnenolone <0.01%, and pregnenolone <0.01%.

### 2.4 Total protein and oxidative stress markers

Total protein concentration in plasma samples was quantified using the Pierce BCA Protein Assay Kit (Thermo Scientific™, Waltham, Massachusetts, USA). Prior to analysis, plasma samples were diluted 1:40 in phosphate-buffered saline (PBS, 10 mM, pH 7.2). Aliquots of 25 μL were dispensed in duplicate into a microplate, followed by the addition of 200 μL of working reagent. Absorbance was recorded at 570 nm using a Victor microplate reader (PerkinElmer, Waltham, MA, USA), and protein concentrations were expressed as mg/mL.

Advanced oxidation protein products (AOPP) levels were determined in plasma samples following the method described by Witko-Sarsat et al. (31). After incubation for 3 min at room temperature, absorbance was measured at 340 nm using a Victor microplate reader (PerkinElmer, Waltham, MA, USA). Results were expressed as nmol AOPP per mg of total protein (nmol AOPP/mg PT).

### 2.5 Glucose and lactate

Plasma glucose and lactate levels were measured using a BIONSEN analyzer (EKF Diagnostics, Cardiff, UK), following the manufacturer’s instructions. Briefly, 20 μL of plasma was used in combination with the EKF pre-filled Glucose/Lactate Hemolyzing Solution (1,000 μL) and measured according to the instrument protocol. Samples falling outside the analytical range of the instrument were excluded from the analysis. Plasma glucose data from the high-density trial were not included in the statistical analysis because a substantial proportion of samples (17/20) fell below the analytical detection range of the instrument despite repeated measurements.

### 2.6 RNA extraction and real-time quantitative PCR

Total RNA was extracted from liver samples using the RNeasy Mini Kit (Qiagen, Hilden, Germany), following the manufacturer’s instructions with on-column DNase digestion. Briefly, approximately 15–20 mg of liver tissue previously preserved in RNAlater was homogenized in RLT buffer supplemented with dithiothreitol (DTT) using a TissueLyser II (Qiagen, Hilden, Germany). Following homogenization and centrifugation, the supernatant was mixed with 70% ethanol and transferred to RNeasy spin columns. Genomic DNA contamination was removed by on-column DNase I treatment with the RNase-Free DNase Set (Qiagen, Hilden, Germany). Subsequent washing steps were performed using RW1 and RPE buffers, and total RNA was finally eluted in RNase-free water.

RNA concentration and purity were assessed using a NanoDrop 2000 spectrophotometer (Thermo Fisher Scientific™, Waltham, MA, USA) by measuring the A260/A280 and A260/A230 absorbance ratios. All RNA samples were considered suitable for downstream RT-qPCR analyses based on their concentration and purity.

First-strand cDNA was synthesized from DNase I-treated total RNA using SuperScript II Reverse Transcriptase (Invitrogen™, Carlsbad, CA, USA) and random hexamer primers. Briefly, RNA samples were first denatured at 65 °C for 10 min in the presence of EDTA, dNTPs, and random hexamers. Reverse transcription was carried out by adding a reaction mix containing First-Strand Buffer, dithiothreitol (DTT), and SuperScript II Reverse Transcriptase. The reaction was incubated at room temperature for 10 min, followed by 50 min at 42 °C for cDNA synthesis, and terminated by heating at 71 °C for 10 min. The resulting cDNA was diluted 1:10 with RNase-free water and stored at −20 °C until further analysis.

Real-time PCR reactions were performed in triplicate using a 7,500 Real-Time PCR System (Applied Biosystems, Life Technologies, Waltham, MA, USA) and analyzed with SDS Software (Applied Biosystems, Life Technologies, Waltham, MA, USA). Relative gene expression analysis was conducted for genes involved in growth (IGF-I) and stress response (HSP70 and CYP1A1). Primer sequences for all target and reference genes were obtained from previously published and validated studies and are reported in Table 1.

**Table 1:** QPCR primers sequences.

Gene	Primer forward (5′–3′)	Primer reverse (5′–3′)	References
β-ACT	CAGCCCTCCTTCCTCGGTAT	AGCACCGTGTTGGCGTACA	(55)
CYP1A1	AGATCTTCCGGCACTCTTCCTTC	GATGTGTCCTTGATCGTGCAGTG	(56)
HSP70	GCACAGAGGGAGAAGATAGCTG	ACCACTTTCTTTTTGTCCTCCTGG	(56)
IGF-I	TGGACACGCTGCAGTTTGTGTGT	CACTCGTCCACAATACCACGGT	(57)

No-template controls were included in each qPCR run to verify the absence of contamination. Amplification specificity was confirmed by melt-curve analysis, which showed a single peak at the expected melting temperature (Tm) for each amplicon. No evidence of primer-dimer formation or non-specific amplification was observed. Expression levels were normalized using *β*-actin (β-ACT) as the reference gene, selected based on previous studies reporting its stable expression in rainbow trout liver, including under stress conditions (37, 38). Relative gene expression was calculated using the ΔΔCt method (39).

### 2.7 Statistical analysis

All statistical analyses were performed using R software (version 4.5.2; R Foundation for Statistical Computing, Vienna, Austria). Data are presented as mean ± standard error (SE).

The normality of data distribution was assessed using the Shapiro–Wilk test, whereas homogeneity of variance was evaluated using Levene’s test. When data met the assumptions of normality and homogeneity of variance, differences in the physiological and biochemical parameters between CTRL and PEC groups were evaluated using a linear mixed-effects model, with treatment as a fixed factor and tank as a random factor.

For gene expression analyses, the mean expression value of the fish sampled from each tank was used as the experimental unit. Owing to the limited number of independent tank replicates, gene expression data were analyzed using either Student’s *t*-test or the Wilcoxon rank-sum test, depending on whether the assumptions of normality were met.

Differences were considered statistically significant at *p* < 0.05.

## 3 Results

### 3.1 Water quality

The physicochemical water quality parameters recorded during the low- and high-density trials have been previously reported in the companion studies by Mirra et al. (36) and are summarized here to provide the environmental context for the biological responses observed in the present study. During the low-density trial, water temperature ranged from approximately 14.1–15.2 °C, dissolved oxygen remained between approximately 7.9 and 8.9 mg L^−1^, while pH gradually decreased from approximately 8.3–7.5 in both the CTRL and PEC systems (36). Ammonia concentrations were initially slightly higher in the PEC tanks but became consistently lower than in the CTRL system from day 8 onwards (0.120 vs. 0.150 ppm at the end of the trial, *p* = 0.053). Nitrite concentrations showed a similar temporal pattern in both groups, although values tended to be lower in the PEC tanks during the first half of the trial (1.41 vs. 1.53 ppm), whereas nitrate concentrations were significantly lower in the PEC system throughout the experiment (*p* < 0.001) (36).

During the high-density trial, water temperature ranged from approximately 13.6–16.2 °C, dissolved oxygen ranged from approximately 7.5–8.3 mg L^−1^, and pH decreased from approximately 8.0–7.1 (40). Fish reared in the PEC system showed significantly lower unionized ammonia (NH₃) concentrations than the CTRL group (0.96 ± 0.20 vs. 1.78 ± 0.20 mg L^−1^, *p* < 0.05), whereas nitrite concentrations did not differ significantly between treatments (2.58 ± 0.29 vs. 2.84 ± 0.29 mg L^−1^). Conversely, nitrate concentrations were significantly higher in the PEC tanks than in the CTRL tanks (61.77 ± 2.14 vs. 53.10 ± 2.14 mg L^−1^, *p* < 0.05) (40).

Titanium was never detected in the water by ICP-MS analysis in either trial, confirming the stability of the TiO_2_ coating throughout the experimental period (36).

### 3.2 Radioimmunoassay (RIA)

No significant differences in cortisol levels were observed between CTRL and PEC groups across all analyzed matrices (plasma, skin mucus, muscle, fins, and scales) in the low-density (LD; 15 kg/m^3^) trial. Cortisol concentrations are reported in Table 2.

**Table 2:** Cortisol levels in adult rainbow trout during the low-density (LD) trial (15 kg/m^3^).

Treatment	Plasma (ng/ml)	Mucus (ng/ml)	Muscle (ng/g)	Fin (ng/g)	Scales (ng/g)
CTRL	1.92 ± 0.29	0.19 ± 0.01	0.39 ± 0.05	0.35 ± 0.04	0.56 ± 0.07
PEC	2.28 ± 0.20	0.19 ± 0.01	0.39 ± 0.03	0.42 ± 0.06	0.53 ± 0.07

Data are expressed as mean ± standard error (SE). *n* = 15 fish per group. Differences between CTRL and PEC were analyzed using a linear mixed-effects model (LMM).

At high stocking density (HD; 30 kg/m^3^), cortisol levels were generally higher compared to the LD trial; however, no significant differences were detected between CTRL and PEC groups in any of the analyzed matrices. Cortisol concentrations are reported in Table 3.

**Table 3:** Cortisol levels in adult rainbow trout during the high density (HD) trial (30 kg/m^3^).

Treatment	Plasma (ng/ml)	Mucus (ng/ml)	Muscle (ng/g)	Fin (ng/g)	Scales (ng/g)
CTRL	3.87 ± 1.07	0.54 ± 0.05	0.48 ± 0.06	1.74 ± 0.16	0.73 ± 0.12
PEC	5.57 ± 0.84	0.73 ± 0.09	0.60 ± 0.08	1.77 ± 0.26	0.91 ± 0.13

Data are expressed as mean ± standard error (SE). *n* = 10 fish per group. Differences between CTRL and PEC were analyzed using a linear mixed-effects model (LMM).

### 3.3 Total protein and oxidative stress markers

At low-density, no significant differences were detected between CTRL and PEC groups for total protein (TP), AOPP levels, or the AOPP/TP ratio (Table 4).

**Table 4:** Total protein (TP) and oxidative stress markers during the low-density (LD) trial (15 kg/m^3^).

Treatment	TP (mg/ml)	AOPP (nmol/ml)	AOPP/TP (ng/g)
CTRL	36.00 ± 1.44	84.90 ± 10.21	2.58 ± 0.32
PEC	34.80 ± 1.59	95.25 ± 8.66	2.64 ± 0.27

Data are expressed as mean ± standard error (SE). *n* = 15 fish per group. Differences between CTRL and PEC were analyzed using a linear mixed-effects model (LMM).

At high-density, TP and AOPP/TP values did not differ significantly between groups (Table 5). However, AOPP levels were significantly higher in the PEC group compared to CTRL (*p* < 0.001).

**Table 5:** Total protein (TP) and oxidative stress markers during the high density (HD) trial (30 kg/m^3^).

Treatment	TP (mg/ml)	AOPP^*^ (nmol/ml)	AOPP/TP (ng/g)
CTRL	42.90 ± 1.45	214.82 ± 22.75	5.00 ± 0.49
PEC	36.74 ± 2.08	352.71 ± 25.35	9.84 ± 0.87

Data are expressed as mean ± standard error (SE). *n* = 10 fish per group. Differences between CTRL and PEC were analyzed using a linear mixed-effects model (LMM). Significant differences are indicated in the table (*p* < 0.001).

### 3.4 Glucose and lactate

At low stocking density (LD; 15 kg/m^3^), no significant differences were observed between CTRL and PEC groups in plasma glucose and lactate levels (Table 6).

**Table 6:** Plasma glucose and lactate during the low-density (LD) trial (15 kg/m^3^).

Treatment	GLU (mg/dL)	LACT (mmol/L)
CTRL	20.31 ± 2.30	8.99 ± 0.46
PEC	25.22 ± 2.34	8.62 ± 0.51

Data are expressed as mean ± standard error (SE). *n* = 15 fish per group. Differences between CTRL and PEC were analyzed using a linear mixed-effects model (LMM).

At high stocking density (HD; 30 kg/m^3^), plasma glucose values were not included in the analysis because multiple samples fell outside the detectable range of the instrument. Lactate levels did not differ significantly between the experimental groups (CTRL 10.89 ± 0.59 mmol/L; PEC 12.53 ± 0.44 mmol/L).

### 3.5 Gene expression

The relative expression of IGF-I (Figure 1A), HSP70 (Figure 1B), and CYP1A1 (Figure 1C) in the liver of adult trout reared at low stocking density (15 kg/m^3^) did not show significant differences between CTRL and PEC groups for any of the analyzed genes.

**Figure 1:**
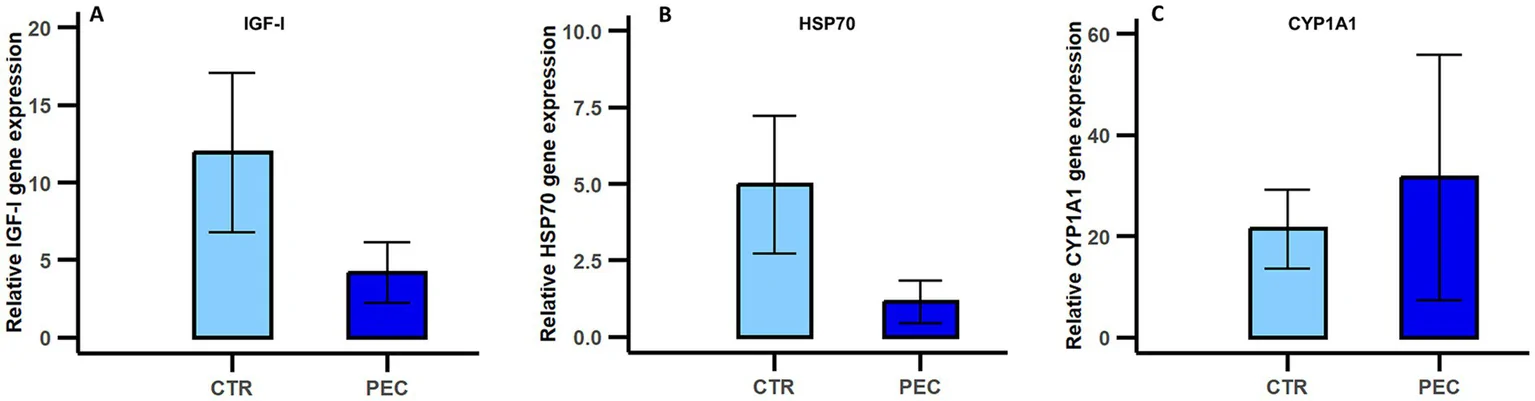
Relative gene expression of IGF-I **(A)**, HSP70 **(B)**, and CYP1A1 **(C)** in the liver of adult rainbow trout reared in CTRL and PEC systems during the low-density (LD) trial (15 kg/m^3^). Six fish per treatment (two fish per tank) were analyzed. Statistical analyses were performed using tank means (*n* = 3 tanks per treatment). Data are presented as mean ± standard error (SE). Differences between groups were analyzed using the Wilcoxon rank-sum test.

The relative expression of the selected genes in the livers of adult trout reared at high stocking density (30 kg/m^3^) is reported in Figure 2. No significant differences were observed between CTRL and PEC groups for IGF-I (Figure. 2A), HSP70 (Figure. 2B), and CYP1A1 (Figure. 2C).

**Figure 2:**
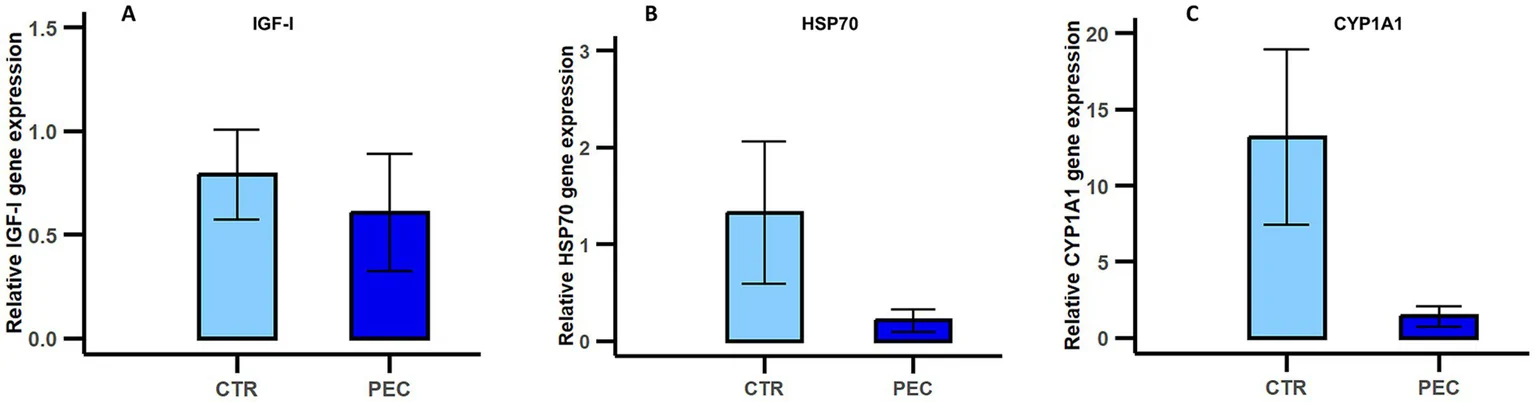
Relative gene expression of IGF-I **(A)**, HSP70 **(B)**, and CYP1A1 **(C)** in the liver of adult rainbow trout reared in CTRL and PEC systems during the high-density (HD) trial (30 kg/m^3^). Six fish from the CTRL group and four fish from the PEC group were analyzed. Statistical analyses were performed using tank means (CTRL: *n* = 3 tanks; PEC: *n* = 2 tanks). Data are presented as mean ± standard error (SE). Differences between groups were analyzed using Student’s *t*-test for IGF-I and CYP1A1 and the Wilcoxon rank-sum test for HSP70.

## 4 Discussion

Photocatalytic systems have gained increasing attention in recent years due to their promising performance in water treatment. In particular, they improve water quality by promoting the oxidation of ammonia and other nitrogenous compounds into less toxic species. Furthermore, their integration with electrocatalysis has been shown to enhance the conversion efficiency of ammonia into molecular nitrogen, representing a valuable strategy for nitrogen removal (14–16). Consistently, improvements in water quality have been reported (33), and preliminary evidence on fish health suggests that PEC systems do not induce evident adverse morphological or biochemical responses, while in some cases even promoting improved physiological conditions compared to control groups (14, 40). Moreover, titanium was never detected in the rearing water by ICP analysis in either the low- or high-density trials (36), indicating the absence of measurable TiO_2_ release from the immobilized photoelectrocatalytic system under the experimental conditions.

In line with these findings, the present study showed no significant differences between CTRL and PEC systems in cortisol, glucose, and lactate levels within either the low- or high-density trial, indicating comparable physiological stress status between the two rearing systems under the respective experimental conditions. Similarly, no differences were observed in the expression of growth- and stress-related genes within each trial, suggesting that the PEC system did not negatively affect molecular responses. Although higher AOPP levels were observed in the PEC group during the high-density trial, cortisol remained within physiological ranges, supporting the absence of severe systemic stress.

Cortisol levels, measured across all analyses matrices, remained within basal ranges reported in the literature. In plasma, mucus, and muscle, which reflect acute stress responses, cortisol concentrations were consistent with typical levels reported for salmonids (in plasma: 0–5 ng/mL, (41); <5 ng/mL, (42); in mucus: 0.2 ng/mL, in muscle: 0.2 ng/g, (18)), indicating no activation of acute stress responses. Likewise, cortisol levels in fin and scales, indicators of chronic stress exposure, were consistent with baseline values (18, 20), supporting the absence of chronic stress under PEC conditions.

Interestingly, lactate and glucose levels exceeded typical reference ranges reported for rainbow trout (43–46). Changes in plasma glucose and lactate levels reflect complex metabolic responses that are not exclusively linked to stress-related endocrine activation (26). Glucose typically increases to meet energetic demands and decreases due to rapid utilization by tissues (26, 47). Lactate accumulation is mainly associated with anaerobic metabolism during conditions of increased energy demand (48). However, this increase was observed in both groups and is likely due to experimental conditions or sampling procedures rather than differences between rearing systems. It is important to consider that cortisol levels remained within basal ranges, which supports the interpretation that fish were not experiencing significant systemic stress. Both glucose and lactate are also influenced by environmental and experimental factors, including handling, sampling procedures, and water conditions (26, 47).

High stocking density is widely recognized as a chronic husbandry stressor in aquaculture, potentially affecting fish welfare through increased crowding, higher metabolic demand, changes in water quality, and altered social interactions (34, 35, 49). Although the present study was not designed to directly compare stocking densities, these mechanisms may contribute to an increased susceptibility to oxidative disturbances under more challenging rearing conditions.

Regarding oxidative stress, AOPP levels were higher in the PEC group under high-density conditions, while the AOPP/TP ratio remained unchanged between groups. Given that the turnover of oxidized proteins is tightly regulated and that oxidative stress can activate adaptive responses, including the induction of heat shock proteins and antioxidant enzymes (50, 51), this pattern may reflect a mild and localized oxidative response rather than a systemic imbalance. Since AOPP is considered an early marker of oxidative protein damage (52, 53), the observed increase could represent an initial oxidative response preceding the activation of broader systemic stress pathways. However, because basal cortisol levels, the AOPP/TP ratio, and the expression of stress-related genes, including HSP70, remained unchanged, AOPP alone is not sufficient to infer the occurrence of a generalized oxidative stress response. Therefore, the biological relevance of this finding should be interpreted with caution, and future studies including a broader panel of oxidative stress biomarkers, particularly antioxidant enzyme activities (e.g., SOD, CAT, and GPx), as well as more comprehensive molecular approaches, are warranted to better characterize the oxidative, immune, and metabolic responses of fish exposed to the PEC system (54).

A limitation of the present study is that the low- and high-density conditions were investigated in two independent trials with different exposure durations (4 and 2 weeks, respectively). Consequently, the present study was not designed to directly compare the effects of stocking density, and the influence of stocking density and exposure time cannot be disentangled. The shorter exposure adopted for the high-density trial was selected as a precautionary measure to minimize prolonged exposure to a husbandry condition known to challenge fish welfare (34, 35). Future studies employing identical exposure periods across stocking densities will be necessary to specifically investigate the effects of stocking density independently of exposure duration.

Overall, the results obtained indicate that the integration of PEC within RAS does not induce adverse stress responses and represents a promising strategy for improving water quality without compromising fish health. Nevertheless, further investigations are warranted to better characterize the observed increase in AOPP levels, as well as to clarify the variability in glucose and lactate levels, particularly in relation to animal handling and management practices.

The experimental conditions, although controlled, do not fully reflect commercial aquaculture settings, where higher stocking densities and more variable environmental conditions may occur and further studies under farm-scale conditions could be interesting to validate the applicability of PEC systems in production scenarios. Moreover, the performance of the PEC system in different water types, such as seawater, remains to be investigated. Finally, while this study focused on biomarkers associated with negative welfare, future research should also include indicators of positive welfare to provide a more comprehensive assessment of fish welfare.

## 5 Conclusion

The results of the present study, complementing previous findings within the same project, provide further insight into the effects of PEC systems on fish stress status and growth. Under the tested experimental conditions, the PEC system did not induce major changes in the measured physiological and molecular stress biomarkers. However, the increase in AOPP under high-density conditions suggests a possible mild oxidative response that warrants further investigation. Overall, these findings, together with the improved water quality and other health-related parameters previously reported, support the potential of PEC technology for application in recirculating aquaculture systems. Nevertheless, further research is needed to optimize the application of PEC systems in aquaculture and to better characterize the role of AOPP and oxidative stress under different farming conditions.

## Data Availability

The raw data supporting the conclusions of this article will be made available by the authors, without undue reservation. Requests to access these datasets should be directed to giuseppe.radaelli@unipd.it.
